# State Medical Association Matters

**Published:** 1904-10

**Authors:** F. E. Daniel

**Affiliations:** President; State Medical Association of Texas; Office of the President; Austin, Texas


### Society Notes.

### State Medical Association Matters.

A copy of the following letter was mailed to each county medical society September 19, 1904:

STATE MEDICAL ASSOCIATION OF TEXAS.

OFFICE OF THE PRESIDENT.

AUSTIN, TEXAS, September 16, 1904.

CIRCULAR LETTER TO COUNTY MEDICAL SOCIETIES.

*To the Officers and Members of* ———— *Co. Medical Society.*

GENTLEMEN: I beg to remind you that the by-laws require that each county society shall have a Committee on Legislation. If not already appointed, I advise that you have a meeting at a very early date and appoint one, and instruct them to personally see each candidate for the Legislature and say that the State Medical Association, now embracing practically the entire regular profession of the State, through its Committee on Public Policy and Legislation (Wilson, Cantrell, Bennett, Daniel and Chase), will ask the next Legislature to enact certain measures in the interest of the public health, and earnestly solicit their support in the interest of those measures. A meeting of the committee will be held here before the election, when it is purposed to determine upon the details of said bills. When this is done a copy of each will be submitted to each county society; but until the committee meets no form of bill can be submitted.

The candidates should be informed that the medical profession, now united, is working *con amore,* in the interest of the public health, the paramount interest of every State and Nation, and that a State Board of Health, with ample authority to formulate and enforce a sanitary code, and the creation of a Bureau of Vital Statistics made effective, as was contemplated by the bill of last session—are the first requisites; and that it is not contemplated to interfere with the quarantine law or to create new offices with large salaries, but to ask for only an appropriation sufficient to make the said board effective.

The creation of a State Board of Health with proper authority will render special legislation unnecessary for many purposes; e. g., prevention of pollution of water supplies, the adulteration of foods, etc., disinfection of premises after infectious diseases, etc. The bill will ask that the constitutional requirement as to State Board of Health and Vital Statistics be put into effect.

A bill to regulate the practice of medicine will probably be presented to correct the clause in the existing bill that unjustly, and in violation of the Constitution, exempts from the requirements of a State license a large class of persons who claim exemption because they do not give drugs. Candidates must be enlightened on this point and made to see that the law grants special privileges to these persons; and that the practice of medicine does not consist alone of giving drugs, and that the courts of many States have ruled that they are practicing medicine in the meaning of the law.

Your society will be informed of the action of the committee, it is hoped, in time to attend to this important matter before election.

Respectfully,

F. E. Daniel,
President.

